# Alkaline hydrolysis to remove potentially infectious viral RNA contaminants from DNA

**DOI:** 10.1186/s12985-016-0552-0

**Published:** 2016-06-04

**Authors:** Karissa A. Lemire, Yelitza Y. Rodriguez, Michael T. McIntosh

**Affiliations:** Foreign Animal Disease Diagnostic Laboratory, NVSL, STAS, VS, APHIS, USDA, Plum Island Animal Disease Center, PO Box 848, Greenport, NY 11944-0848 USA

## Abstract

**Background:**

Diagnostics and research of high-consequence animal disease agents is often limited to laboratories with a high level of biosecurity that restrict the transport of biological material. Often, sharing of DNA with external partners is needed to support diagnostics, forensics, or research. Even in the absence of virus, RNA from positive-sense single stranded RNA (+ssRNA) viruses that may contaminate otherwise purified DNA preparations continues to pose a threat due to its potential to be infectious via direct translation to yield viral proteins. While the risk of animal infection or accidental reconstitution and release of a virus from RNA is very low, the high impact of an animal disease event associated with the accidental release of some + ssRNA viruses, such as classical swine fever or foot-and-mouth disease viruses, necessitates the precaution of having procedures to ensure the complete inactivation of viruses and + ssRNA viral genomes. RNA and DNA are differentially susceptible to enzymatic degradations; however, such procedures are susceptible to unintended DNA damage and/or failure due to enzyme or cofactor instabilities. Therefore, we describe the development and verification of a robust and simple chemical and physical method to selectively degrade RNA from purified DNA preparations. The procedure employs incubation of DNA in 0.25 N sodium hydroxide at 65 °C for 1 h followed by neutralization and boiling for 10 min to hydrolyze contaminating RNA and inactivate animal disease viruses from DNA preparations. Additional critical quality control elements include use of a synthetic control RNA (SCR) and an SCR-specific real-time RT-PCR to track effectiveness of the procedure in a parallel treated control sample, and a pH check of reagents to ensure proper neutralization of alkaline conditions.

**Results:**

The new procedure reduced intact RNA beyond the limit of detection by realtime RT-PCR and inactivated viruses by in vitro culture infectivity assays.

**Conclusions:**

Treated DNA, while denatured, remains suitable for most common molecular biology procedures including PCR, transformation of *E. coli*, and molecular sequencing. The procedure ensures not only the inactivation of a variety of viruses but also the degradation through hydrolysis of potentially contaminating infectious + ssRNA viral genomes.

## Background

Positive-sense single stranded RNA (+ssRNA) viruses of agricultural and economic importance include agents such as foot-and-mouth disease virus (FMDV) and classical swine fever virus (CSFV). FMDV is a highly contagious disease of cloven-hoofed animals including cattle, swine, sheep, goats, and various wildlife species. FMDV is a non-enveloped virus belonging to the genus *Aphthovirus* in the family *Picornaviridae*. There are seven known serotypes of FMDV including types A, O, C, Asia1, South African Territories (SAT) 1, SAT2, and SAT3 [1]. Although mortality in adult animals is low, its wide distribution, high transmission rates, and broad host range in livestock make it one of the leading threats to animal agriculture, reviewed in [2]. CSFV is another highly contagious disease of livestock, affecting specifically swine and wild boars, and belonging to the genus *Pestivirus* in the family *Flaviviridae* [3]. Although there is only one known serotype of CSFV, several strains ranging in virulence have been identified, some with high mortality rates, reviewed in [4]. Both FMDV and CSFV can result in high-consequence animal disease outbreaks with significant economic losses in livestock industries and trade and are reportable to the World Organization of Animal Health (OIE). Consequently, diagnostics and research on such high-consequence animal disease pathogens is typically limited to laboratories with a high level of biosecurity and restricted permissible transport of biological materials out of the laboratory. Such precautions include the export of purified nucleic acids.

Even in the absence of virus, RNA from + ssRNA viruses continues to pose a threat to agricultural industries due to its potential to be infectious via direct translation yielding viral proteins [5]. For instance, transfection studies employing DNA contaminated with RNA from + ssRNA viruses can inadvertently result in the expression of infectious virus. Often, however, the sharing of DNA with external partner laboratories is needed to support diagnostics, forensics, or research and development activities. For example, non-infectious DNA samples, such as human DNA associated with forensic samples from an animal disease investigation, might need to be shared with a criminal investigation laboratory. Alternately, a portion of complementary DNA (cDNA) or DNA from a virus may need to be sent, either directly or cloned into plasmid DNA, to another laboratory as an experimental reagent. Because these DNA materials originate from a biocontainment laboratory that works with + ssRNA viruses of high-consequence, trace amounts of virus or even viral RNA present concerning sources of potentially infectious contamination that must be mitigated prior to the transport of DNA outside of biocontainment.

**Fig. 1:**
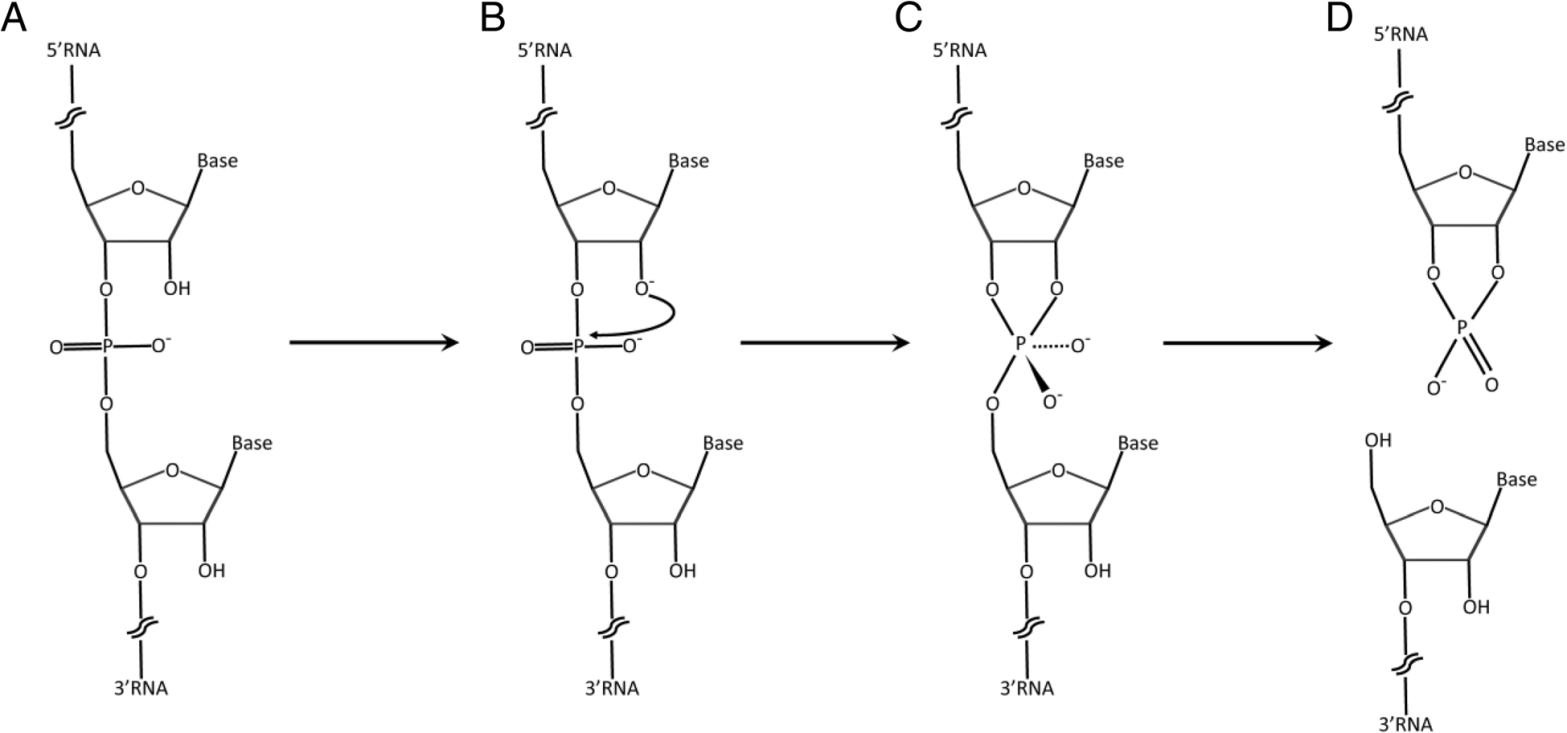
Schematic of RNA degradation under extreme alkaline pH conditions. **a** The phosphopentose backbone of RNA is shown. **b** The 2’ hydroxyl (OH) of RNA becomes deprotonated under extreme alkaline conditions leading to a nucleophilic attack on the 5’ phosphate (PO_4_) of the adjacent nucleotide. **c** An intermediate 2’,3’ cyclic phosphate is formed resulting in, **d** cleavage of the phosphopentose backbone of RNA. The resultant 2’,3’-cyclic phosphate may be further hydrolyzed to 2' or to 3' phosphate leaving RNA fragments or free ribonucleotides with 5’-OH and 3’-phosphates

Due to subtle differences in the structures of RNA and DNA, including a hydroxyl group on the 2’-carbon atom of the phosphopentose backbone of RNA, RNA reactivity and stability under certain conditions is fundamentally different than that of DNA [6]. For example in the early 1900’s, shortly after the discovery of RNA and DNA, the instability of RNA and contrasting stability of DNA under basic conditions was recognized. This fundamental biochemical property of RNA formed the basis of widely established alkaline treatment methods for the purification of free or partially hydrolyzed ribonucleotides from RNA [7], and modern methods of degrading RNA in labeling or stripping of DNA microarrays or in advanced molecular sequencing protocols [8–11]. In contrast, the stability of ssDNA under alkaline conditions formed the basis for the alkaline lysis method of plasmid DNA purification from bacteria [12]. RNA is uniquely unstable in alkaline conditions because bases can easily deprotonate the hydrogen from the hydroxyl group on the 2’-carbon atom (Fig. 1). This deprotonation causes the oxygen to become negatively charged leading to a nucleophilic attack on the adjacent phosphate atom leading to the cleavage of the phosphopentose backbone of RNA. The resultant 2',3'-cyclic phosphate is further hydrolyzed to 2' or to 3' phosphate leaving RNA fragments or free ribonucleotides with 5’-OH and 3’-phosphates, depending on the level of degradation (Fig. 1). Conversely the lack of a 2’-OH in DNA prevents cleavage of the phosphate backbone making DNA relatively stable at high pH. As this autocatalytic degradation is a property of the RNA phosphopentose backbone itself, the use of alkaline pH combined with heat treatment has been used to selectively degrade RNA from RNA:DNA or RNA:cDNA hybrids [10, 11, 13].

While RNA and DNA are also differentially susceptible to enzymatic degradations, such procedures may cause unintended DNA damage and/or fail due to enzyme or cofactor instabilities. This paper aims to describe the development and verification of a robust and simple chemical method to selectively degrade RNA. The procedure is demonstrated to be useful in the inactivation of viruses and the removal of RNA, including potentially infectious viral + ssRNA, from a purified DNA sample. Extreme alkaline (0.25 N NaOH at pH > 12) and heat treatment were chosen as the basis of the new DNA safety treatment method based on the well-established property of RNA degradation and ssDNA stability under alkaline conditions, previously established sensitivity of high-consequence animal disease viruses to heat inactivation by boiling for 5 min, and documented inactivation of FMDV and CSFV at alkaline pH. In this regard, FMDV is inactivated at pH levels less than 6 and greater than 9 [14], and CSFV is known to be inactivated at pH levels less than 3 and greater than 11 [15]. While alkaline or heat treatment are both individually expected to denature dsDNA, this procedure, based on the verification studies presented herein and earlier studies on heat inactivation of animal disease viruses [16, 17], may form the basis for inactivation of potentially contaminating viruses and the robust degradation of viral + ssRNA from purified DNA. Following alkaline and heat treatment, the denatured DNA remains suitable for recovery by precipitation and manipulation in routine molecular biology procedures such as transformation of *E.coli* and molecular sequencing.

## Results

### DNA integrity and loss of RNA

Integrity of DNA, such that safety treated DNA remains intact, unmodified, and suitable for manipulation in most routine molecular biology procedures, was an important criteria in the development of the alkaline and heat DNA safety treatment procedure. While, alkaline and heat treatment are both individually expected to denature dsDNA, confirming minimal single stranded nicks and double strand breaks was deemed important for downstream applications such as bacterial transformation of plasmids, PCR, and DNA sequencing.

DNA integrity and loss of RNA was evaluated by agarose gel electrophoresis, rRT-PCR, and sequencing. Various samples including, plasmid and/or CSFV and African swine fever virus (ASFV) PCR amplified cDNA or DNA, CSFV RNA, and/or synthetic control RNA (SCR) spiked samples after NaOH and heat treatment, heat treatment alone, or no treatment as control were evaluated.

Table 1 addresses initial studies aimed at verification of RNA degradation while maintaining plasmid DNA integrity using a NaOH and heat treatment incubation time of 30 min as compared to the later determined optimal treatment time of 60 min. CSFV RNA and SCR spiked samples containing plasmid DNA were tested in various conditions ranging from untreated control samples to safety treated plasmid DNA samples containing SCR as an internal control and CSFV RNA as a simulated contaminant (Table 1). All positive controls (untreated, heat only treatment, or 250 mM NaCl containing samples) for both SCR RNA and CSFV RNA tested positive by rRT-PCR for their respective targets (Table 1). All negative controls (water or samples with no target added) tested negative for SCR and CSFV RNA by rRT-PCR (Table 1). All safety treated samples tested negative by SCR and CSF rRT-PCRs for their respective targets with the exception of sample 4, suggesting that the time of incubation for NaOH and heat treatment could be further optimized. Plasmid DNA integrity was maintained with some slight nicking and denaturation to ssDNA as visualized by agarose gel electrophoresis (data not shown).

**Table 1:** Verification of RNA degradation while maintaining plasmid DNA integrity

Sample ID	Heat	Plasmid	CSFV RNA	SCR	NaOH	HCl	TE	PCR	DNA
1- SCR RNA	x	0 μl	0 μl	26 μl	0 μl	0 μl	260 μl	SCR+, CSF-	NA
2- Plasmid DNA	x	130 μl	0 μl	0 μl	0 μl	0 μl	156 μl	SCR-, CSF-	+
3- SCR RNA/Plasmid DNA	x	130 μl	0 μl	26 μl	0 μl	0 μl	130 μl	SCR+, CSF-	+
4- SCR RNA treated	x	0 μl	0 μl	26 μl	60 μl	60 μl	140 μl	SCR+, CSF-	NA
5- Plasmid DNA treated	x	130 μl	0 μl	0 μl	60 μl	60 μl	36 μl	SCR-, CSF-	+^a^
6- SCR RNA/Plasmid DNA treated	x	130 μl	0 μl	26 μl	60 μl	60 μl	10 μl	SCR-, CSF-	+^a^
7- CSF RNA	x	0 μl	10 μl	0 μl	0 μl	0 μl	276 μl	SCR-, CSF+	NA
8- CSF RNA/SCR RNA	x	0 μl	10 μl	26 μl	0 μl	0 μl	250 μl	SCR+, CSF+	NA
9- CSF RNA/SCR RNA/ Plasmid DNA	x	130 μl	10 μl	26 μl	0 μl	0 μl	166 μl	SCR+, CSF+	+
10- CSF RNA/Plasmid DNA	x	130 μl	10 μl	0 μl	0 μl	0 μl	140 μl	SCR-, CSF+	+
11- CSF RNA treated	x	0 μl	10 μl	0 μl	60 μl	60 μl	130 μl	SCR-, CSF-	NA
12- CSF RNA/SCR RNA treated	x	0 μl	10 μl	26 μl	60 μl	60 μl	156 μl	SCR-, CSF-	NA
13- CSF RNA/SCR RNA/ Plasmid DNA treated	x	130 μl	10 μl	26 μl	60 μl	60 μl	0 μl	SCR-, CSF-	+^a^
14- CSF RNA/Plasmid DNA treated	x	130 μl	10 μl	0 μl	60 μl	60 μl	260 μl	SCR-, CSF-	+^a^
15- SCR RNA post pH normalization	x	0 μl	0 μl	26μl^b^	60 μl	60 μl	146 μl	SCR+, CSF-	NA
16- Plasmid DNA post pH normalization	x	130 μl^b^	0 μl	0 μl	60 μl	60 μl	36 μl	SCR-, CSF-	+
17- CSF RNA post pH normalization	x	0 μl	10 μl^b^	0 μl	60 μl	60 μl	130 μl	SCR-, CSF+	NA
18- CSF RNA/SCR RNA post pH normalization	x	0 μl	10μl^b^	26μl^b^	60 μl	60 μl	156 μl	SCR+, CSF+	NA
19- CSF RNA/SCR RNA/Plasmid DNA post pH normalization	x	130μl^b^	10μl^b^	26μl^b^	60 μl	60 μl	0 μl	SCR+, CSF+	+
20- CSF RNA undilute control		0 μl	2.5 μl	0 μl	0 μl	0 μl	0 μl	SCR-, CSF+	NA
21- CSF RNA diluted control		0 μl	10 μl	0 μl	0 μl	0 μl	276 μl	SCR-, CSF+	NA
22- SCR RNA diluted control		0 μl	0 μl	26 μl	0 μl	0 μl	260 μl	SCR+, CSF-	NA
23- SCR RNA undilute control		0 μl	0 μl	2.5 μl	0 μl	0 μl	0 μl	SCR+, CSF-	NA
24- CSF positive amplification control		0 μl	2.5 μl	0 μl	0 μl	0 μl	0 μl	SCR-, CSF+	NA
25- No template control for PCR		0 μl	0 μl	0 μl	0 μl	0 μl	H_2_O	SCR-, CSF-	NA

Samples, containing various combinations of SCR RNA, CSF RNA, and pGEM Teasy Vector plasmid, treatment conditions, and results for verification of RNA degradation and maintenance of plasmid DNA integrity are listed. NaOH and heat treatment incubation time used in this initial study was 30 min. ^a^Slight nicking or denatured to ssDNA was observed as compared to untreated control plasmid. ^b^Reagent added post pH neutralization as a positive control sample. NA indicates not analyzed; x indicates 60 °C incubation and 10’ boiling steps were included

**Fig. 2:**
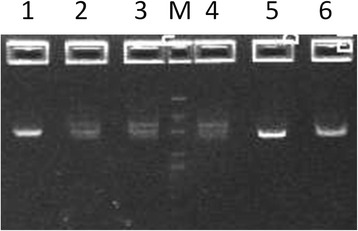
Plasmid DNA integrity following alkaline and heat treatment. Imaging of pGEM Teasy plasmid DNA samples as visualized by gel electrophoresis. Safety treated DNA samples are shown following incubation in 0.25 N NaOH at 65 °C for 30, 45 and 60 min followed by neutralization of pH and incubation at 100 °C for 10 min (lanes 2, 3 and 4, respectively). Controls consisted of heat only treated DNA (lane 1, 60 min at 65 °C and 10 min at 100 °C in 250 mM NaCl and TE pH 8.0), or untreated plasmid in TE or 250 mM NaCl (lanes 5 and 6, respectively). Molecular weight markers are 10 kbp, 4 kbp, 2 kbp, 800 bp and 400 bp (lane M)

Table 2 and Fig. 2 address safety treatment optimization studies aimed at verifying SCR degradation while maintaining viral and plasmid DNA integrity using NaOH and heat treatment incubation times of 30, 45 and 60 min. CSFV cDNA, plasmid and ASFV DNA samples were tested in various conditions ranging from untreated control samples to safety treated DNA samples all containing SCR internal controls (Table 2). All positive controls (untreated, heat only treatment or diluted samples) tested positive by SCR rRT-PCR. SCR was not degraded beyond the level of rRT-PCR detection during the safety treatment process for the 30 and 45 min incubations at 65 °C in 0.25 N NaOH as indicated by positive SCR rRT-PCR results (Table 2). All SCR RNA samples incubated for 60 min at 65 °C tested negative for SCR RNA by rRT-PCR (Table 2).

**Table 2:** Optimization of RNA hydrolysis while maintaining viral and plasmid DNA integrity

Sample ID	Heat (min)	DNA	SCR RNA	NaOH	HCl	TE	SCR PCR	ASF PCR	CSF PCR	DNA integrity
1- ASF DNA control	0	35 μl	7 μl	0 μl	0 μl	30 μl	+	+	NA	+
2- CSF DNA control	0	35 μl	7 μl	0 μl	0 μl	30 μl	+	NA	+	+
3- Plasmid DNA control	0	35 μl	7 μl	0 μl	0 μl	30 μl	+	NA	NA	+
4- ASF DNA heated	30	35 μl	7 μl	0 μl	0 μl	30 μl	+	+	NA	+
5- CSF DNA heated	30	35 μl	7 μl	0 μl	0 μl	30 μl	+	NA	+	+
6- Plasmid DNA heated	30	35 μl	7 μl	0 μl	0 μl	30 μl	+	NA	NA	+
7- ASF DNA treated	30	35 μl	7 μl	15 μl	15 μl	0 μl	-	+	NA	+
8- CSF DNA treated	30	35 μl	7 μl	15 μl	15 μl	0 μl	+	NA	+	+
9- Plasmid DNA treated	30	35 μl	7 μl	15 μl	15 μl	0 μl	+	NA	NA	+^a^
10- ASF DNA treated	45	35 μl	7 μl	15 μl	15 μl	0 μl	-	Inc^b^	NA	+
11- CSF DNA treated	45	35 μl	7 μl	15 μl	15 μl	0 μl	+	NA	+	+
12- Plasmid DNA treated	45	35 μl	7 μl	15 μl	15 μl	0 μl	-	NA	NA	+^a^
13- ASF DNA treated	60	35 μl	7 μl	15 μl	15 μl	0 μl	-	Inc^b^	NA	+
14- CSF DNA treated	60	35 μl	7 μl	15 μl	15 μl	0 μl	-	NA	+	+
15- Plasmid DNA treated	60	35 μl	7 μl	15 μl	15 μl	0 μl	-	NA	NA	+^a^
16- SCR in TE	0	0 μl	7 μl	0 μl	0 μl	65 μl	+	NA	NA	NA
17- SCR in salt	0	0 μl	7 μl	15μl^c^	15μl^c^	35 μl	+	NA	NA	NA
18- SCR undilute control	0	0 μl	2.5 μl	0 μl	0 μl	0 μl	+	NA	NA	NA

Samples containing various combinations of ASF DNA, CSF cDNA, and plasmid DNA, under various treatment conditions. All samples contained SCR, and results for verification of SCR degradation and maintenance of plasmid DNA integrity are listed. NaOH and heat treatment incubation time used in this study range from 0 to 60 min. Following heat treatment for various times at alkaline pH, all samples were boiled for 10 min at neutral pH. ^a^Slight nicking or denatured ssDNA was observed as compared to untreated control plasmid. ^b^Inconclusive rPCR result (Inc) due to high concentration of DNA template. ^c^Neutralized salt solution (equal parts 1 N NaOH and 1 N HCl) reagent used as a positive control. NA indicates not analyzed

All ASFV DNA samples, controls and treatment samples retained intact DNA. This was verified by ASF rPCR in all samples, with the exception of samples 10 and 13 that yielded anomalous rPCR amplification dynamics due to high ASFV DNA template concentrations (Table 2). All CSFV cDNA samples likewise tested positive by CSF rRT-PCR, indicating DNA integrity was maintained throughout the process (Table 2). Plasmid DNA integrity was maintained with some slight nicking and/or denaturation of ssDNA as indicated by supercoiled untreated plasmid DNA and less sharp banding with slightly slow mobility on agarose gel electrophoresis (Fig. 2). No significant difference in the integrity of CSFV cDNA, plasmid, or ASFV DNA was apparent regardless of the time of incubation for alkaline hydrolysis; therefore, 60 min was selected as the optimal time of alkaline and heat hydrolysis to ensure complete degradation of all RNA (Table 2 and Fig. 2).

### Effects on bacterial transformations

Transformation efficiency of alkali and heat treated DNA was evaluated using *E. coli* strains DH5 alpha, JM109, and BL21. Alkali and heat safety treated pBlueScript II plasmid, or untreated plasmid as control, were used to transform bacteria using a variation of the heat shock method [18]. Each bacterial strain readily yielded transformants, albeit efficiencies were reduced by 6.6 to 54.8 fold as compared to transformations with untreated plasmid. Blue white colony screening by alpha complementation was used with the DH5 alpha strain to demonstrate proper expression and function of the plasmid encoded *LacZ* gene product before and after alkali and heat safety treatment. In this regard, all bacterial DH5 alpha colonies were blue suggesting a lack of significant mutation leading to loss in expression or function of the alpha peptide of β-galactosidase.

### Sequencing of DNA post alkaline and heat hydrolysis of RNA

**Fig. 3:**
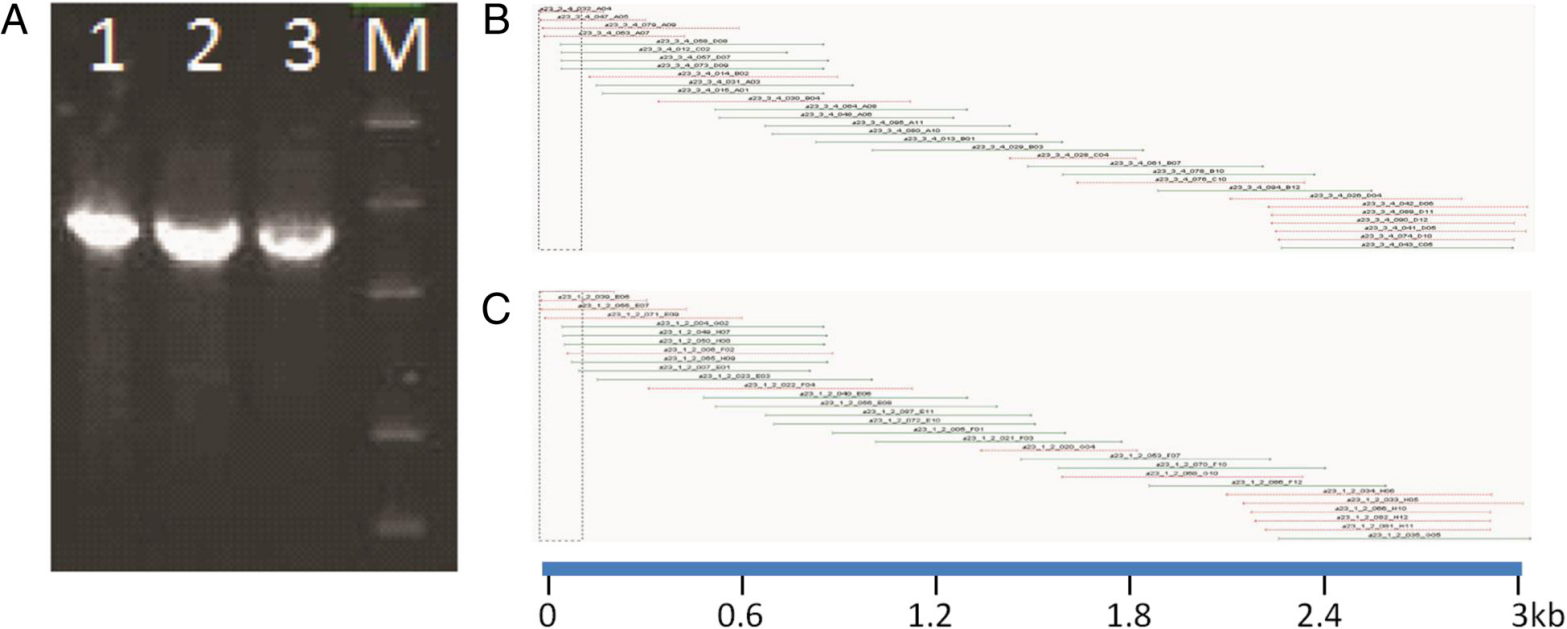
Effects of safety treatment on FMDV cDNA by gel electrophoresis and sequencing. **a** RT-PCR amplified FMDV A23 Iraq P1 coding region observed by gel electrophoresis. Untreated FMDV P1 cDNA in TE pH 8.0 (lane 1), in 250 mM NaCl (lane 2), and safety treated FMDV P1 cDNA (lane 3) are shown. **b** Sequencing contig assembly chart for alkaline and heat treated FMDV P1 cDNA with sense (green) and antisense (red) sequencing reads shown. **c** Sequencing contig assembly chart for untreated FMDV P1 cDNA with scale bar. Molecular weight markers are 10 kbp, 4 kbp, 2 kbp, 800 bp and 400 bp (lane M)

FMDV cDNA integrity was analyzed by sequencing of the P1 region of the FMDV genome. Maintenance of DNA integrity is important to ensure successful performance of downstream applications. Table 3 and Fig. 3 address downstream application studies aimed at verification of DNA integrity by FMDV sequencing of the P1 region of the genome. FMDV cDNA samples were tested in various conditions ranging from untreated control samples to safety treated DNA samples incubated at the optimized incubation time of 60 min (Table 3). All FMDV cDNA samples, including untreated, heat only treated, and alkali and heat treated samples were successfully sequenced at comparable coverage (Fig. 3). All high quality DNA sequences appeared to be identical in comparisons to untreated control FMDV cDNA.

**Table 3:** Effects of safety treatment on FMDV P1 cDNA sequencing applications

Sample ID	Heat	SCR	NaOH	HCl	TE/ H_2_O	P1 Sequence
1- FMD P1 cDNA heated	x	0 μl	0 μl	0 μl	37 μl	+
2- FMD P1 cDNA + SCR heated	x	7 μl	0 μl	0 μl	30 μl	+
3- FMD P1 cDNA treated	x	0 μl	15 μl	15 μl	7 μl	+
4- FMD P1 cDNA + SCR treated	x	7 μl	15 μl	15 μl	0 μl	+
5- FMD P1 cDNA diluted in H_2_O		0 μl	0 μl	0 μl	37 μl	+
6- FMD P1 cDNA diluted salt		0 μl	15 μl^a^	15 μl^a^	7 μl	+
7- FMD P1 cDNA control		0 μl	0 μl	0 μl	0 μl	+

Samples containing various combinations of FMDV cDNA, SCR, and/or treatment conditions. FMDV sequencing results of 35 μl FMDV cDNA spanning the P1 coding region of the genome were determined to verify cDNA integrity under different treatment conditions. NaOH and heat treatment incubation time used in this study was 60 min as optimized in previous studies. ^a^A neutralized salt solution containing equal parts of NaOH and HCl was added instead of individual base and acid treatments to serve as a positive control. X indicates 65 °C for 60 min and 10 min boiling steps were included, and + indicates positive sequencing results

### Inactivation of viruses post alkaline and heat hydrolysis of RNA or boiling

Virus isolation was performed to ensure inactivation of FMDV and CSFV during the NaOH and heat DNA safety treatment process which included a final 10 min boiling step. Table 4 addresses studies aimed at verification of the inactivation of infectious FMDV and CSFV by heat only treatment (65 °C for 1 h followed by boiling for 10 min) or the complete NaOH and heat safety treatment using infectivity assays in primary LK or the SK-6 cell line, respectively. FMDV and CSFV spiked samples were tested under various conditions ranging from untreated, heat only and NaOH and heat safety treatment (Table 4). All positive controls (untreated and unheated, undilute or virus in diluents: TE, salt, or water) tested positive for cytopathic effect for FMDV and by immunohistochemical staining for CSFV (Table 4). All heat only treated samples tested negative for cytopathic effect for FMDV and by immunohistochemical staining for CSFV, suggesting that heat alone was sufficient in making FMDV and CSFV non-infectious (Table 4). All safety treated and heated samples tested negative for cytopathic effect for FMDV and by immunohistochemical staining for CSFV, indicating that the entire safety treatment process (chemical treatment and heat treatment in combination) was successful at inactivating both FMD and CSF viruses (Table 4).

**Table 4:** Sample composition, treatment and virus infectivity

Sample	TCID_50_	NaOH	65 °C Incubation	NaCl	Post Neutralization Boil	VI	FMD rRT-PCR	CSF rRT-PCR
1- FMDV	6.53x10^4^	-	-	-	-	+	NT	NT
2- CSFV	4.11x10^5^	-	-	-	-	+	NT	NT
3- FMDV	3.51x10^4^	-	-	-	-	+	NT	NT
4- CSFV	2.21x10^5^	-	-	-	-	+	NT	NT
5- FMDV	3.51x10^4^	-	-	0.25 N^a^	-	+	NT	NT
6- CSFV	2.21x10^5^	-	-	0.25 N^a^	-	+	NT	NT
7- FMDV	3.51x10^4^	-	-	-	-	+	NT	NT
8- CSFV	2.21x10^5^	-	-	-	-	+	NT	NT
9- FMDV + SCR	2.81x10^4^	-	60 min	-	10 min	-	NT	NT
10- CSFV + SCR	1.77x10^5^	-	60 min	-	10 min	-	NT	NT
11- FMDV+ SCR	2.81x10^4^	0.25 N	60 min	0.25 N	10 min	-	NT	NT
12- CSFV + SCR	1.77x10^5^	0.25 N	60 min	0.25 N	10 min	-	NT	NT
13- NEG TE	0	-	-	-	-	-	NT	NT
14- NEG Salt	0	-	-	0.25 N^a^	-	-	NT	NT
15- NEG Salt	0	-	-	0.25 N^a^	-	-	NT	NT
16- NEG MEM	0	-	-	-	-	-	NT	NT
17- FMDV + SCR	3.98x10^6^	-	-	-	-	+	+	NT
18- FMDV + SCR	3.98x10^6^	-	60 min	-	-	+	+	NT
19- FMDV + SCR	3.98x10^6^	-	-	-	5 min	-	+	NT
20- FMDV + SCR	3.98x10^6^	-	-	-	10 min	-	+	NT
21- FMDV + SCR	3.98x10^6^	0.25 N	60 min at 25 °C	0.25 N	-	-	-	NT
22- FMDV + SCR	3.98x10^6^	0.25 N	60 min	0.25 N	10 min	-	-	NT
23- TE	0	-	-	-	-	-	-	NT
24- CSFV + SCR	2.23x10^4^	-	-	-	-	+	NT	+
25- CSFV + SCR	2.23x10^4^	-	60 min	-	-	+	NT	+
26- CSFV + SCR	2.23x10^4^	-	-	-	5 min	-	NT	+
27- CSFV + SCR	2.23x10^4^	-	-	-	10 min	-	NT	+
28- CSFV + SCR	2.23x10^4^	0.25 N	60 min at 25 °C	0.25 N	-	-	NT	+
29- CSFV + SCR	2.23x10^4^	0.25 N	60 min	0.25 N	10 min	-	NT	-
30- TE	0	-	-	-	-	-	NT	-

Samples containing various combinations of FMDV, CSFV, SCR, and treatment conditions are shown. Virus isolation (VI) results are presented to verify inactivation of infectious FMDV and CSFV under various conditions. The NaOH and heat treatment incubation time used in this study was the optimized 60 min incubation at 65 °C. ^a^Neutralized salt solution was added instead of NaOH. NT indicates not tested; − indicates negative rRT-PCR result or reagent or condition not added; + indicates positive rRT-PCR result

Indeed, earlier studies performed at the USDA, Foreign Animal Disease Diagnostic Laboratory (FADDL) previously established boiling as a method to inactivate a number of animal disease viruses (Table 5; J. A. House, et al., unpublished observations). Viruses tested included African horse sickness virus (serotype 4), ASFV (Brazil strain), FMDV (O1 Campos), lumpy skin disease virus (Ismalia strain), porcine parvovirus (NVSL strain), rinderpest virus (Kabete O strain), swine vesicular disease (UK 27/72 strain), vesicular exanthema of swine (serotype A 48), and vesicular stomatitis virus (serotype Indiana 1). Each high titer virus sample was treated by boiling for 5 or 10 min in culture medium containing 2 to 5 % fetal bovine serum. Infectivity was assessed by in vitro cultivation and titering in Vero, swine kidney cell line (IBRS-2), fetal bovine lung (FBL) or swine testicular cells (ST) depending on the virus. In all instances, boiling for both 5 and 10 min were found to be effective at eliminating infectivity as assayed by in vitro infectivity assays. This data formed the basis for retaining a 10 min boiling step following the NaOH and heat hydrolysis of RNA as an added measure to ensure the inactivation of the above listed animal disease viruses.

**Table 5:** Results of inactivation of selected viruses by boiling

Virus Species	Virus Genus (Family)	Cell Line	Days of Incubation	Virus Titers and Boiling Times (Log base 10 of TCID_50_ ml^−1^)		
				0 min	5 min	10 min
African horse sickness virus (Serotype 4)	*Orbivirus* *(Reoviridae)*	Vero	10	7.7	nvd	nvd
African swine fever virus (Brazil strain)	*Asfivirus (Asfarviridae)*	Vero	10	5.9	nvd	nvd
Foot-and-mouth disease virus (O1 Campos)	*Apthovirus* *(Picornaviridae)*	IBRS-2	3	7.5	nvd	nvd
Lumpy skin disease virus (Ismalia strain)	*Capripoxvirus* *(Poxviridae)*	FBL	13	7.9	nvd	nvd
Porcine parvovirus (NVSL strain)	*Protoparvovirus* *(Parvovirinae)*	ST	10	5.9	nvd	nvd
Rinderpest virus (Kabete O strain)	*Morbillivirus* *(Paramyxoviridae)*	Vero	9	6.4	nvd	nvd
Swine vesicular disease (UK 27/72 strain)	*Enterovirus* *(Picornaviridae)*	IBRS-2	3	6.8	nvd	nvd
Vesicular exanthema of swine (Serotype A 48)	*Vesivirus* *(Caliciviridae)*	Vero	3	8.9	nvd	nvd
Vesicular stomatitis virus (Serotype Indiana 1)	*Vesiculovirus* *(Rhabdoviridae)*	Vero	3	5.8	nvd	nvd

No virus detected (nvd); African green monkey kidney cell line (Vero); swine kidney cell line (IBRS-2); fetal bovine lung (FBL); swine testicular cell line (ST); J. A. House, et al., unpublished observations

### Efficacy of alkaline and heat hydrolysis treatment

After development and formalization of the protocol, the DNA safety treatment procedure was conducted on 15 separate occasions for a total of 80 individual DNA samples. SCR rRT-PCR product was detected in two samples near the limit of detection (Ct = 38.04 and 38.15) yielding a specificity of 97.5 % (95 % CI ± 3.42 %) for hydrolysis of SCR beyond the limit of rRT-PCR detection. Sensitivity of the SCR rRT-PCR was 100 % (99 % CI ± 0 %). DNA safety treatment of the two samples in which SCR was detectable by rRT-PCR was repeated with complete elimination of detectable SCR. Hence to reduce risk to a minimal level, safety treated DNA should not be released from biocontainment without a parallel control sample having a negative SCR rRT-PCR result.

## Discussion

The high impact of an animal disease event associated with the accidental release of high consequence animal disease viruses necessitates the precaution of having procedures to ensure the complete inactivation of viruses and + ssRNA viral genomes. In the studies presented here, boiling alone for five minutes was demonstrated to inactivate African horse sickness virus, African swine fever virus, foot-and-mouth disease virus, lumpy skin disease virus, porcine parvovirus, swine vesicular disease virus, vesicular exanthema of swine virus, classical swine fever virus, and vesicular stomatitis virus (Tables 4 and 5), and alkaline and heat treated samples spiked with FMDV and CSFV likewise demonstrated loss of virus infectivity in vitro (Table 4). The presented procedure relies not only on hot alkaline conditions (0.25 N NaOH and 65 °C for 1 h) for the degradation of all RNA, presumably to free ribonucleotides with 5’-hydroxyls and 3’-phosphates, but also on the additional step of boiling at 100 °C for 10 min after pH has been neutralized to between pH 7 and 8. As even a few breaks in viral genomic RNA are likely to prevent reconstitution of infectious virus, the standard of complete loss of rRT-PCR detectable SCR may appear to be an overly stringent assessment for verification of the DNA safety treatment procedure. Admittedly, the risk of animal infection or accidental reconstitution and release of a virus from RNA contaminants of purified DNA is extremely low. Published examples of + ssRNA viruses reconstituted in animals are few and typically involve direct injection of highly purified and abundant in vitro transcribed viral RNA within a cellular transfection medium such as lipofectamine [19, 20]. In vitro, also using cell transfection methods, recovery of virus from RNA is more common; though, RNA needs to be of high quality, presumably with few or no breaks within protein coding or regulatory elements [19, 21, 22]. It is hoped that the stringent measure of RNA degradation employed herein will add confidence in the safety of exported DNA from laboratories that work with highly infectious animal disease viruses of national and international economic impact such as FMDV.

It is also hoped that the alkaline and heat treatment procedure will improve the quality of DNA over other methods of DNA safety treatment such as RNAse A treatment [23]. In this regard, NaOH and heat treatment are expected to provide more effective degradation of protein and/or lipid encapsulated RNA as well as RNA within an RNA:RNA or RNA:DNA duplex. Indeed, similar treatments with NaOH and heat have been found to be effective in removal of RNA from cDNA preparations used in transcriptome analyses using quantitative RT-PCR or microarrays [10, 11].

## Conclusions

Although denaturation of dsDNA to ssDNA and/or nicking of supercoiled plasmid DNA to relaxed covalently closed structures is anticipated from both alkaline and heat treatment, results of all experiments presented in this summary demonstrated maintenance of integrity of the DNA suitable for routine molecular biology manipulations including PCR, rPCR, DNA sequencing, and bacterial transformations. However, evaluation of safety treated DNA for the potential accumulation of mutations due to error-prone DNA repair following introduction of treated DNA to eukaryotic cells or to diverse strains of *E.coli* has yet to be undertaken. One should anticipate a potential one to two log reduction in efficiency of bacterial transformations using alkaline and heat treated plasmids, and while treated DNA may be used directly for transforming chemically competent *E. coli*, it must be desalted prior to bacterial transformation by electroporation. Incorrect neutralization of the alkaline conditions represents a notable pitfall in the DNA safety treatment procedure. This may lead to autocatalytic depurination, DNA nicking, dsDNA breaks or complete degradation of DNA if the neutralization step is incorrectly performed, resulting in acidic condition. To mitigate this, the procedure includes a pH check of quality critical NaOH and HCl reagents. Despite these potential technical pitfalls, alkaline and heat hydrolysis to remove RNA from purified DNA represents an attractive option for the safe sharing of DNA between biocontainment laboratories that work with + ssRNA viruses.

## Methods

### NaOH and heat safety treatment of purified DNA

Plasmid and other DNA samples ≤ 5 mg/ml buffered with ≤ 100 mM Tris pH 8.0 or standard TE pH 8.0 are suitable for alkaline and heat safety treatment. The DNA safety treatment is conducted in three phases. First, potential contaminating viral or bacterial agents and viral positive sense genomic RNA that may be infectious is destroyed by 1 h heat (65 °C and alkali treatment at 0.25 N sodium hydroxide (pH >12). Second, sample pH is neutralized with equi-normal HCl to ensure usefulness of DNA in downstream molecular biology applications. Third, any potential contaminating virus present is inactivated by boiling at 100 °C for 10 min. Samples are slow cooled to room temperature for 20 min to renature DNA prior to being stored frozen. Treated sample tubes must remain closed after the neutralization step and throughout the boiling step until exported from the biocontainment lab to prevent potential post-treatment contamination.

Prior to the treatment procedure, reagents are quality control checked for proper neutralization. Briefly, 2.33 parts by volume of TE buffer (pH 8.0), 1 part by volume 1 N NaOH and 1 part by volume of 1 N HCl are combined and mixed gently. The solution is tested with a pH meter or pH strips to ensure a proper neutralized pH between 7 and 8.

SCR was developed as an internal control to ensure proper degradation of contaminating RNA before exportation from a containment laboratory. The SCR was spiked into a parallel sample that was 1/5 the volume of the primary sample. Treatment of this parallel control sample is performed in the same manner with the same proportions of NaOH and HCl reagents and is tested by rRT-PCR for SCR detection along with an untreated SCR positive control to ensure proper performance of the procedure and effective degradation of any potentially contaminating RNA.

### Cells and viruses

All cell lines were supplied by the Reagent and Vaccine Services Section (RVSS) of the USDA National Veterinary Services Laboratories’ (NVSL) FADDL located at the Plum Island Animal Disease Center (PIADC). Swine kidney-6 (SK-6), primary lamb kidney (LK), and African green monkey (Vero) cells were grown in Eagle’s minimum essential medium (EMEM) supplemented with 10 % fetal bovine serum and incubated at 37 °C, 5 % CO_2_ , and 100 % humidity. Earlier studies (J. A. House, et al., unpublished observations) conducted in 1994 (Table 5), to assess infectivity of various viruses after boiling for 5 and 10 min, utilized Vero, swine kidney cell line (IBRS-2), and primary fetal bovine lung (FBL), and swine testicular cell line (ST) cultured under similar conditions. Virus inocula including African horse sickness virus (serotype 4), African swine fever virus (Brazil strain), FMDV (O1 Campos), lumpy skin disease virus (Ismalia strain), porcine parvovirus (NVSL strain), rinderpest virus (Kabete O strain), swine vesicular disease (UK 27/72 strain), vesicular exanthema of swine (serotype A 48), and vesicular stomatitis virus (serotype Indiana 1) were treated by boiling for 5 or 10 min in culture medium containing 2 to 5 % fetal bovine serum.

Viruses were supplied by the RVSS repository of the FADDL. FMDV stocks were propagated in primary LK cells. A cell monolayer greater than 80 % confluent was infected with FMDV C4 Tierra del Fuego (1.0 x 10^7^ TCID_50_/ml) or FMDV O Venezuela (2.51 x 10^5^ TCID_50_/ml) and observed for cytopathic effects (CPE) for a maximum of 72 h. Flasks were placed in a −70 °C freezer when greater than 80 % of the monolayer displayed CPE. Virus infected LK cultures were freeze-thawed, harvested, and stored frozen at −70 °C. Virus titrations were performed in 96-well plates using the Spearman- Kärber 50 % tissue culture method [24]. CSFV stocks were propagated in SK-6 cells. A cell monolayer greater than 70 % confluent was infected with CSFV Brescia (1.12 x 10^6^ TCID_50_/ml) or CSFV Kanagawa (1.58 x 10^6^ TCID_50_/ml), incubated for 72 h, and subjected to a freeze thaw prior to harvesting and storing at −70 °C. Virus titrations were performed in 24-well plates and immunohistochemistry was performed using the VECTASTAIN® ABC-AP KIT according to the manufacturer instructions. ASFV Killean III (6.31 x 10^7^ TCID_50_/ml) strain was propagated in swine macrophage cells and observed for hemadsorption (rosette formation) for a maximum of 10 days. Flasks were freeze thawed, aliquotted, and frozen at −70 °C until use. The 50 % tissue culture infectious dose using the Spearman-Kärber method was used to calculate titrations [24].

ASFV was grown in primary cultures of peripheral blood macrophages collected from a donor pig. Swine macrophages were isolated from fresh whole swine blood using a Ficoll-paque gradient and centrifugation to separate the three phases: plasma, white blood cells (buffy coat) and red blood cells. The buffy coat fraction was then isolated and washed several times in RPMI/ 1 % antibiotic media and plated in PRIMARIA™ coated culture flasks. Macrophages were concentrated and replated prior to infection and each lot was isolated fresh for each infection. All cell lines were sterility and virus sensitivity tested.

### Virus isolation of safety treated samples

Virus isolation was performed to ensure inactivation of FMDV and CSFV during the NaOH and heat DNA safety treatment process. Starting titers for different stocks of FMDV O Venezuela V3205 and CSFV Kanagawa V2861 were 2.51 x 10^5^ TCID_50_/ml or 3.55 x 10^7^ TCID_50_/ml and 1.58 x 10^6^ TCID_50_/ml or 1.99 x 10^5^ TCID_50_/ml, respectively. NaOH and/or heat treatments were performed as described below. Heating steps included incubation at 65 °C for 1 h and boiling at 100 °C for 10 min. Various concentrations of virus in diluents, TE or 0.23 M NaCl (salt), representing the final salt concentration of neutralized NaOH treated samples, were tested with or without heating. FMDV or CSFV infectivity was assessed by in vitro cultivation in susceptible cells in EMEM supplemented with 4 % FBS at 37 °C, 5 % CO_2_, and 100 % humidity. FMDV infectivity was assessed by cultivation in primary LK cells in 25 cm^2^ polystyrene tissue culture treated flasks. LK cells, approximately 80 % confluent, were inoculated with 260 μl of each FMDV containing sample and observed every 24 h post inoculation for evidence of cytopathic effects (CPE). CPE negative flasks were observed for a total of 96 h followed by freeze-thaw and second passage on LK cells for an additional 96 h to ensure the absence of infectious FMDV. CSFV infectivity was assessed by cultivation in SK-6 cells. SK-6 cells, approximately 70 % confluent, were inoculated with 100 μl of each CSFV containing sample in duplicate wells of a 24-well polystyrene tissue culture plate. At 72 h post inoculation, monolayers were fixed in 60 % acetone and 40 % methanol at −20 °C for 10 min and stained using the VECTASTAIN® ABC-AP KIT according to the manufacturer instructions. Stained monolayers were evaluated microscopically to determine presence or absence of infectious CSFV.

### Nucleic acids

RNA extractions for FMDV and CSFV were performed using the RNeasy® Mini Kit (Qiagen, Valencia, CA, USA) and DNA extractions for ASFV were performed using the DNeasy® Blood and Tissue Kit (Qiagen, Valencia, CA, USA) according to manufacturer’s instructions. The pGEM-T Easy vector plasmid (Promega Corp.) was propagated in overnight cultures of transformed JM109 or TOP10 *E.coli* in LB medium containing 100 μg ml^−1^ ampicillin. The pBlueScript II plasmid (Agilent Technologies) was similarly propagated in *E.coli* DH5 alpha. Plasmids were purified using the Qiagen QIAquick Spin Miniprep PCR Purification Kit as per manufacturer’s instructions. Plasmid preparations were quantified on a NanoDrop 2000 spectrophotometer and stored frozen at −30 °C.

An internal 199 bp synthetic control RNA (SCR), designed partially from the bacteriophage Q beta, was purchased from IDT (sequence: UCUUAAGUCGAUAAAUGCUUAUUGCUCUCUUAGCACUGGGUUUUACAAACCUGUGGAUGGCGUGAUAGUUGGCUUUUGGCGCGAUCCAUCCAUCCUUUGCACGCCGUGGGACGACUUCACUGCGAGUCCUGCCACGGAGUCCUAUUCAAGCCGUGAUAGUCGUUCCUCGUGCUUAGUAACUAAGGAUGAAAUGCAUGUC). The SCR was incorporated into parallel safety treatments of split samples to ensure proper degradation of contaminating RNA before exportation from a containment laboratory. Specific primer and probe binding sites were included in the sequence of the SCR for rRT-PCR detection. The SCR was diluted in TE buffer (pH 8.0) and used at a concentration of 6 x 10^−6^ ng ml^−1^, which yields a cycle threshold (Ct) value of 30 in rRT-PCR.

Qualitative evaluations of plasmid DNA and PCR products before and after treatments were performed electrophoresis in 2 % agarose gels using the E-Gel Electrophoresis System (Thermo Fisher Scientific),

### Bacterial transformations

Transformation efficiencies for alkali and heat treated DNA were evaluated using competent *E. coli* DH5 alpha (Invitrogen-Thermo Fisher Scientific), JM109 and BL21(DE3) pLys S (Promega), and 60 ng alkali and heat treated pBlueScript II plasmid (Agilent Technologies) or untreated plasmid as control. Bacterial transformations were performed using a variation of the heat shock method described previously [18], prior to plating on LB agar containing 100 μg ml^−1^ ampicillin, 80 μg ml^−1^ X-gal, and 50 mM IPTG (Teknova). Expression and function of the *LacZ* gene product from pBlueScript II was evaluated by blue white screening of DH5 alpha colonies, and transformation efficiencies were calculated in colony forming units per μg of plasmid.

### Real-time and conventional PCR and RT-PCR

Real-time RT-PCR was performed to optimize the NaOH safety treatment method and to evaluate SCR degradation, CSF RNA degradation, CSF DNA integrity, and ASF DNA integrity after NaOH and heat safety treatment, heat treatment alone, and no treatment as a control.

RNA samples were amplified using the GeneAmp® EZ rTth RNA PCR kit (Life Technologies, Grand Island, NY, USA) and a SmartCycler II (Cepheid, Sunnyvale, CA) with automatic background subtraction on, or using the TaqMan® Fast Virus 1-Step Master Mix (Life Technologies, Grand Island, NY, USA) and an Applied Biosystems® 7500 Real-Time PCR System (Life Technologies, Grand Island, NY, USA). The SCR RT-PCR reactions each consisted of 0.2 μM of each primer, 0.1 μM of FAM/TAMRA probe, 5 μl of 5x GeneAmp® EZ Buffer, 5 mM of Mn(OAc)_2_, 1.2 mM of dNTP mix, 1 μl of rTth DNA polymerase, and 2.5 μl of RNA template in a final reaction volume of 25 μl. The thermal profile consisted of reverse transcription at 60 °C for 10 min, followed by 45 cycles of 95 °C for 2 s and 60 °C for 30 s. Primer and probe sequences include SCR forward primer: 5’-ACTGGGTTTTACAAACCTGTGA -3’, SCR reverse primer: 5’- TCCGTGGCAGGACTCGC -3’, and SCR probe: 6FAM-TCCTTTGCACGCCGTGGGAC-TAMRA. A result cut-off, above a manual fluorescence threshold of 30, was defined as positive at Ct ≤ 40, inconclusive at Ct >40, and negative if no Ct was observed.

After discontinuation of the GeneAmp® EZ rTth RNA PCR reagents by Life Technologies, a brief methods comparison study was performed using the TaqMan® Fast Virus 1-Step Master Mix (Life Technologies, Grand Island, NY, USA) to ensure reagent sensitivity similar to the tested method. SCR rRT-PCR consisted of 0.2 μM of each primer, 0.1 μM of FAM/TAMRA probe, 6.25 μl of 4× 1-Step Master Mix and 2.5 μl sample in a final reaction volume of 25 μl. The thermal profile consisted of reverse transcription at 50 °C for 5 min and initial denaturation at 95 °C for 20 s, followed by 45 cycles of 95 °C for 15 s and 60 °C for 1 min.

CSFV RNA and CSFV DNA spiked pGEM Teasy vector plasmid DNA samples were amplified after NaOH and heat treatment, heat treatment alone, and no treatment as a control. Samples were amplified using the GeneAmp® EZ rTth RNA PCR kit (Life Technologies, Grand Island, NY, USA). The CSF rRT-PCR reactions each consisted of 0.2 μM of each primer, 0.1 μM of FAM/MGB probe, 5 μl of 5x GeneAmp® EZ Buffer and 2.5 μl sample in a final reaction volume of 25 μl. The thermal profile consisted of reverse transcription at 60 °C for 10 min, followed by 45 cycles of 95 °C for 2 s and 60 °C for 30 s [25].

After discontinuation of the GeneAmp® EZ rTth RNA PCR reagents by Life Technologies, subsequent FMDV and CSFV RNA samples were amplified using the Path-ID™ Multiplex One-Step Kit (Life Technologies, Grand Island, NY, USA). FMD rRT-PCR reactions consisted of 0.2 μM of each primer, 0.1 μM of FAM/TAMRA probe [26], 12.5 μl of 2x Multiplex RT-PCR buffer, 2.5 μl of 10x Multiplex enzyme mix and 2.5 μl sample in a final reaction volume of 25 μl. The thermal profile consisted of reverse transcription at 48 °C for 10 min, 95 °C for 10 min, followed by 45 cycles of 95 °C for 2 s and 60 °C for 40 s using the FAST mode on the Applied Biosystems® 7500 Real-Time PCR System (Life Technologies, Grand Island, NY, USA). CSF rRT-PCR reactions consisted of 0.2 μM forward primer, 0.4 μM reverse primer, 0.2 μM of FAM/MGB probe [25], 12.5 μl of 2x Multiplex RT-PCR buffer, 2.5 μl of 10x Multiplex enzyme mix and 2.5 μl sample in a final reaction volume of 25 μl using the same thermal profile and platform as described above for FMD rRT-PCR.

ASF DNA spiked pGEM Teasy vector plasmid DNA samples were amplified after NaOH treatment and heat treatment, heat treatment alone, and no treatment as a control. Samples were amplified using the TaqMan® EZ RT-PCR kit (Life Technologies, Grand Island, NY, USA). The ASF qPCR reactions consisted of 0.3 μM of each primer, 0.2 μM of FAM/MGB probe, 5 μl of 5x TaqMan® EZ Buffer and 2.5 μl sample in a final reaction volume of 25 μl. The thermal profile consisted of 45 cycles of 95 °C for 2 s and 60 °C for 30 s [27]. All rRT-PCR reactions were performed on the SmartCycler II PCR platform (Cepheid, Sunnyvale, CA, USA). Conventional RT-PCR of the 3 kb capsid coding P1 region of the FMDV genome was performed as previously described [28].

### FMDV cDNA sequencing

The FMDV P1 RT-PCR product was purified using a QIAquick® PCR Purification kit (Qiagen) according to manufacturer’s instructions. Purified FMDV cDNA was quantified using a NanoDrop 2000 spectrophotometer (Fisher Scientific, Pittsburg, PA, USA) and sequenced in 10 μl reactions containing 2 μl of BigDye® Terminator v3.1 (Applied Biosystems), 5 pmol of primer, and 15 ng of purified RT-PCR product as previously described [28]. Thermal cycling conditions consisted of 35 cycles of 10 s at 96 °C, 5 s at 50 °C, followed by 4 min at 60 °C. Sequencing products were purified on a Kingfisher 96 magnetic particle processor (Thermo Fisher Scientific) using a high-throughput the CleanSEQ kit (Agencourt). Nucleotide sequences were resolved using a 3730XL DNA sequencer (Applied Biosystems), and sequence contigs for each sample were compiled using the Sequencher® software (Gene Codes).

## Abbreviations

+ssRNA, positive - sense single stranded RNA; ASFV, African swine fever virus; CSF, classical swine fever virus; dsDNA, double-stranded DNA; FBL, fetal bovine lung; FMDV, foot-and-mouth disease virus; IBRS-2, swine kidney cell line; rRT-PCR, real-time Reverse-transcriptase polymerase chain reaction; SCR, synthetic control RNA; ssDNA, single-stranded DNA; ST, swine testicular cell line; Vero, African green monkey kidney cell line; VI, virus isolation
